# Generation of individualized immunocompatible endothelial cells from HLA-I-matched human pluripotent stem cells

**DOI:** 10.1186/s13287-022-02720-7

**Published:** 2022-02-02

**Authors:** Chanchan Song, Linli Wang, Qingyang Li, Baoyi Liao, Weihua Qiao, Qiang Li, Nianguo Dong, Liangping Li

**Affiliations:** 1 Institute of Clinical Oncology, Research Center of Cancer Diagnosis and Therapy, and Department of Clinical Oncology, The First Affiliated Hospital of Jinan University, Guangzhou, China; 2 Guangzhou Future Homo Sapiens Institute of Biomedicine and Health (GFBH), Guangzhou, China; 3 Guangzhou Regenerative Medicine Research Center, Future Homo Sapiens Institute of Regenerative Medicine Co., Ltd (FHIR), Guangzhou, China; 4 Department of Cardiovascular Surgery, Union Hospital, Tongji Medical College, Huazhong University of Science and Technology, Wuhan, China

**Keywords:** Allogeneic iPSCs, Endothelial cells, Alloresponse, HLA-I modification

## Abstract

**Background:**

Endothelial cells (ECs) derived from human-induced pluripotent stem cell (iPSC) are a valuable cell resource for cardiovascular regeneration. To avoid time-consuming preparation from primary autologous cells, the allogeneic iPSC-ECs are being expected to become “off-the-shelf” cell products. However, allorejection caused by HLA mismatching is a major barrier for this strategy. Although the “hypoimmunogenic” iPSCs could be simply generated by inhibition of HLA-I expression via β-2 microglobulin knockout (B2M KO), the deletion of HLA-I expression will activate natural killer (NK) cells, which kill the HLA-I negative cells. To inhibit NK activation, we proposed to generate HLA-matched iPSCs based on patient’s HLA genotyping by HLA exchanging approach to express the required HLA allele.

**Methods:**

To establish a prototype of HLA exchanging system, the expression of HLA-I molecules of iPSCs was inhibited by CRISPR/Cas9-mediated B2M KO, and then HLA-A*11:01 allele, as a model molecule, was introduced into B2M KO iPSCs by lentiviral gene transfer. HLA-I-modified iPSCs were tested for their pluripotency and ability to differentiate into ECs. The stimulation of iPSC-EC to allogeneic T and NK cells was detected by respective co-culture of PBMC-EC and NK-EC. Finally, the iPSC-ECs were used as the seeding cells to re-endothelialize the decellularized valves.

**Results:**

We generated the iPSCs only expressed one HLA-A allele (HLA-A *11:01) by B2M KO plus HLA gene transfer. These HLA-I-modified iPSCs maintained pluripotency and furthermore were successfully differentiated into functional ECs assessed by tube formation assay. Single HLA-A*11:01-matched iPSC-ECs significantly less induced the allogeneic response of CD8+ T cell and NK cells expressing matched HLA-A*11:01 and other HLA-A,-B and -C alleles. These cells were successfully used to re-endothelialize the decellularized valves.

**Conclusions:**

In summary, a simple HLA-I exchanging system has been created by efficient HLA engineering of iPSCs to evade both of the alloresponse of CD8+ T cells and the activation of NK cells. This technology has been applied to generate iPSC-ECs for the engineering of cellular heart valves. Our strategy should be extremely useful if the “off-the-shelf” and “non-immunogenic” allogeneic iPSCs were created for the common HLA alleles.

**Supplementary Information:**

The online version contains supplementary material available at 10.1186/s13287-022-02720-7.

## Background

Endothelial cells (ECs) lining the inner surface of blood vessels, lymphatic vessels, and heart valve leaflets are essential for the maintenance of integrity and homeostasis of the entire blood and lymph circulation [1]. Disturbances of EC functions are connected to various diseases such as atherosclerosis, hypertension, and valve disorders. [2].

In vitro-cultured ECs have been widely used to model vascular pathology, uncover novel drug targets, and develop promising cell therapies [3–6], e.g., vascular and heart valve tissue engineering [6–8]. The huge value of ECs in basic research and clinical applications demands the production of scalable quantities of the well-defined ECs in laboratory. Current major approaches are to obtain the mature ECs or endothelial progenitors from autologous or allogeneic individuals [9–11]. From an immunological perspective, ECs should be prepared from autologous cells to avoid immune rejection of allograft. However, primary autologous ECs have limited proliferation capacity in vitro and are especially difficult to obtain from older individuals or critically ill patients [12].

In contrast, human pluripotent stem cells (PSCs), such as embryonic stem cells (ESCs) and induced pluripotent stem cells (iPSCs), possess ability of unlimited proliferation and of differentiation into any somatic cell types [13, 14], including ECs. Consequently, PSCs, especially iPSCs, are attractive cell sources for the clinical-scale preparation of ECs [15]. The preparation of autologous iPSCs is complicated, time-consuming and costly for every patient; therefore, allogeneic cells are inevitable choice for the development of iPSC-based regenerative cell therapy.

However, the mismatching of HLA molecules between cells of donor and recipient will induce a potent immune rejection response, in which the recipient T cells recognize and reject allogeneic donor cells with nonself determinants presented by HLAs [16]. HLA molecules are divided into two classes: HLA class I (HLA-I) and HLA class II (HLA-II) [17]. HLA-I molecules (HLA-A, -B, and -C) are expressed on all nucleated cells and display peptides from endogenous and exogenous antigens to CD8+ cytotoxic T cells, while HLA-II molecules (HLA-DP, -DQ, and -DR) are almost exclusively expressed in antigen-presenting cells to present antigens to CD4+ T cells. HLA alleles are highly diverse in human population [18], so that the different HLA molecules present a various group of peptides to form nonself determinants on cell surface.

HLA-I molecules serve as major determinants on grafted somatic cells for the allogeneic immune response of T cells. To overcome the challenges of HLA mismatching, several strategies have been developed to inhibit alloresponse by downregulation of HLA-I expression. As B2M proteins stabilize the HLA structure by forming a heterodimer with HLA-I heavy chains and are essential for HLA-I presentation on the cell surface; hence, deletion of the B2M gene via various approaches: homology targeted disruption, TALEN, CRISPR/Cas9 gene editing or RNA interference (RNAi) had been performed to directly suppress all of HLA-I cell surface expression and to prevent an immune recognition by cytotoxic CD8+ T cells [19–23]. However, HLA-I depletion will activate NK cells through the missing-self response, so that graft of donor cells fails [24].

To solve this dilemma, we proposed that the manipulation of HLA-I genes on the basis of recipient HLA genotyping could create HLA-matched “non-immunogenic” iPSCs for individualized allogeneic grafts. In this study, we report that an HLA exchanging system was established to generate HLA-I-matched iPSCs by the B2M KO plus B2M-HLA fusion gene transfer for any HLA-I type to solve alloresponse of T cells to allogeneic cell grafts and simultaneously suppress the NK activation caused by endogenous HLA-I downregulation via B2M KO.

## Materials and methods

### Cell lines, vectors and mice

Human iPSC lines: LHPb-YaabC55 (C55), LHPb-YaabC56 (C56) and LHPa-MaaaC10 (C10) were derived from human urine-derived cells (hUC) and provided by Guangzhou Future Homo Sapiens Institute of Biomedicine and Health (GFBH) [25]; UH1-F M1 derived from human cord blood CD34+ cells and human ES cell lines (H1, H9 and HN4) were obtained from the Chinese Academy of Sciences; HEK-293T, and human umbilical vein endothelial cell (HUVEC) cell lines were from ATCC. pMD2G (#12259) and psPAX2 (#12260) were from Addgene. The female NCG and nude mice (6–8 weeks) were purchased from Guangdong Medical Laboratory Animal Center and fed in a specific pathogen-free environment at the Experimental Animal Center of Jinan University.

### Cell culture

To grow PSC cell lines, all solutions (medium and buffer) must be pre-warmed at room temperature until no longer cool to touch. The PSCs were routinely maintained in the defined stem cell medium BioCISO (OSINGLAY Investigator, BC-PM001) on 6-well plates pre-coated with 1:100 Matrigel (Corning, 354277) at 37 °C for 1 h. For passaging, cells were dissociated with 0.5 mM EDTA (Invitrogen, 15575020) for 10–15 min and re-plated at the ratio of 1:4–1:6 in fresh BioCISO medium supplemented with 10 μM ROCK inhibitor Y-27632 (SelleckChem, S1049) (BioCISO+Y-27632). The culture medium was changed daily.

HEK-293T and HUVEC lines were maintained in DMEM basic medium (Gibco, 10569010) supplemented with 10% FBS (PAN, P160204). The iPSC-ECs were maintained in StemPro-34 SFM (Gibco, 10639011) supplemented with 50 ng/mL hVEGF-A (StemCell, 78073). All cell lines were cultured at 37 °C in 5% CO_2_ in cell incubator.

### Embryoid body (EB) formation

C55 cell line was differentiated into ectoderm, mesoderm, and endoderm in vitro following the protocol reported previously [25]. The differentiated cells were collected and evaluated the expression of marker genes in the three germ layers (7 markers for endoderm: AFP, FOXA2, SOX17, FGF8, GATA4, cytokeratin 8 and cytokeratin18; 5 for mesoderm: MSX1, Tbx5, GATA6, DESMIN and IGF2; 4 for ectoderm: MAP2, SOX1, PAX6 and GFAP) by quantitative reverse transcription PCR (qRT-PCR).

### Quantitative reverse transcription polymerase chain reaction

Total RNA was isolated using RNAiso Plus (Takara), and M-MLV Reverse Transcriptase (Takara) was used to synthetize cDNA. mRNA expression levels were determined using SYBR Premix Ex Taq™ (Takara). Reactions were performed in triplicate using a LightCycler 480II/96 system (Roche, Switzerland). mRNA expression was normalized to the housekeeping gene GAPDH.

### CRISPR/Cas9 gene editing of *B2M*

To establish HLA-I negative iPSCs, we designed CRISPR/Cas9 B2M gene editing experiment to target “GAGTAGCGCGAGCACAGCTA” DNA sequence of human *B2M* gene. Briefly, 7.5 μg Cas9 protein (Invitrogen, A36498) and 50 pmol B2M sgRNA were mixed to form 12 μL B2M ribonucleoprotein complex (B2M-RNP) and added into 100 μL Nucleofector buffer (Lonza). 4 × 10^5^ C55 iPSCs were transfected with the above B2M-RNP/Nucleofector solution by electroporator 2B Nucleofector with A-023 program. After electroporation, cells were transferred to Matrigel-coated 6-well plates and cultured in BioCISO+Y-27632 medium for 3–5 days. HLA-I expression was detected by staining with anti-HLA-ABC antibodies (Biolegend, 311406). One week later, HLA-ABC negative iPSCs were sorted by FACS AriaII (BD) into Matrigel-coated 24-well plates and grown in BioCISO+Y-27632 medium. Single cell clones of B2M KO iPSCs (C55-B2M^*KO*^) were isolated by limiting dilution cloning (LDC).

### Lentiviral vector-mediated HLA gene transfer

To introduce exogenous HLA-I gene into C55-B2M^*KO*^, we constructed lentiviral HLA-A*11:01 expression vector based on CSII-EF-MCS-IRES2. cDNAs of human *B2M* and HLA-A*11:01 were fused together via (Gly4Ser)3 linker [31] to form 1436 bp DNA fragment and inserted into the NotI and BamHI sites of CSII-EF-MCS-IRES2 vector to construct CSII-EF-hB2M-A11.

Gene transfer of HLA-A*11:01 to C55-B2M^*KO*^ was carried out by lentiviral transduction. Briefly, a total of 1 × 10^6^ HEK-293T cells were seeded in each well of a 6-well plate and grown overnight to 70–80% confluence. Two micrograms of each plasmid (pMD2G, psPAX2, and CSII-EF-hB2M-A11) was mixed with lipofectamine 3000 (Invitrogen, L3000015) for 20 min. The DNA-liposome mixture was gently added to HEK-293T cells in the 6-well plate. After 6–8 h, the culture medium was replaced with 2 mL fresh medium per well. Forty-eight hours later, the hB2M-A11 lentiviral supernatant was harvested. Two  milliliters of single cell suspension of 1 × 10^6^ C55-B2M^*KO*^ was mixed with 2 mL lentiviral supernatant (1:1 dilution) plus 4 μg/mL protamine sulfate in a 6-well plate and incubated at 37 °C overnight. Cell medium was refreshed daily. Ten days later, cells were dissociated with 0.5 mM EDTA and cloned by LDC to obtain pure HLA-I positive iPSCs (C55-A11).

### Analysis for karyotyping and pluripotency of HLA-I-modified iPSCs

To characterize the genomic integrity of iPSCs during manipulation of HLA-I modification, we performed karyotyping, teratoma formation and short tandem repeat (STR) DNA analysis.

For karyotyping analysis, 6 × 10^6^ iPSCs were treated with 20 ng/mL colchicine (Beyotime, ST1173) for 100 min. Preparation of metaphase chromosomes of iPSCs was performed according to standard methods [26]. The slides were stained with Giemsa for G-banding karyotyping. The Ikaros karyotyping system was used to analyze karyotypes.

Teratoma formation assay: 8 × 10^5^ C55-A11 iPSCs were mixed with Matrigel at a 1:1 ratio and transplanted into the forelimb muscle of 3 female NCG mice intramuscularly. Eight weeks later, teratomas were analyzed by the hematoxylin and eosin (H&E) staining of paraffin-embedded sections.

The STR DNA analysis was used to confirm whether the original and modified iPSCs were derived from the same individual and performed by IGEbio.

### EC differentiation from parent and HLA-I-modified iPSCs

To differentiate parent and HLA-I-modified iPSCs into ECs, we chose a priming-induction protocol modified from previous protocol [27]. Briefly, iPSCs were dissociated with 0.5 mM EDTA and incubated in plates coated with 1:30 growth-factor-reduced Matrigel (Corning, 356230) at a density of 1 × 10^4^ cells/cm^2^ in BioCISO+Y-27632 medium. After 24 h, the medium was replaced with Priming Medium, consisting of N2B27 medium (1:1 mixture of DMEM/F-12/GlutaMAX (Gibco, 10565018) and Neurobasal media (Gibco, 21103049) supplemented with 1 × N2 (Gibco, 17502048) and 1 × B27(Gibco, 17504044)) with 8 μM CHIR99021 (MCE, HY-10182) and 25 ng/mL rhBMP4 (Peprotech, 120-05ET). After 3 days, the Priming Medium was replaced by EC induction medium, consisting of StemPro-34 SFM medium (Gibco, 10639011) supplemented with 200 ng/mL hVEGF-A (Stemcell, 78073) and 2 µM forskolin (Sigma-Aldrich, F6886). The induction medium was renewed one day later. On Day 6 of differentiation, ECs were dissociated with Accutase (Stemcell, 07922) and bulk cultured on human fibronectin (Corning, 356008)-coated (1:40 diluted) 75 T cell culture flask at a density of 2.5 × 10^4^ cells/cm^2^ in EC expansion medium, consisting of StemPro-34 SFM supplemented with 50 ng/mL hVEGF-A. EC expansion medium was replaced every 3 days. EC differentiation was monitored by flow cytometry with anti-huCD144-PE (Biolegend, 348506) and anti-huKDR-APC (Biolegend, 359916).

### CD31 immunofluorescent analysis

2 × 10^4^ cells (iPSC-ECs or HUVECs) were seeded on a 25-mm coverslip and cultured overnight. Cells were fixed with 4% paraformaldehyde for 20 min, washed twice with PBS, and incubated in blocking buffer (PBS + 3% BSA) for 30 min, and then stained with 1:1000 anti-huCD31 antibody at 4 °C overnight. After washing three times with blocking buffer, the cells were then incubated with 1:100 Cy3-conjugated goat anti-rabbit IgG second antibody for 50 min at RT. After washing twice, cells were then incubated in DAPI for 10 min at RT, washed twice, and incubated in anti-fluorescence quencher for image documentation.

### Tube formation in vitro

To measure the capillary-like formation of the iPSC-ECs, we performed tube formation experiments on the surface of Matrigel. Seventy-five microliters of Matrigel per well was added to 96-well plate and incubated for 1 h at 37 °C to allow the gel to solidify. 2 × 10^4^ ECs per well were then seeded onto the matrix and cultured in EGM-2 (LONZA, CC-3162) supplemented with 3 ng/mL bFGF (Peprotech, 100-18B) for 18 h at 37 °C and 5% CO_2_. HUVEC served as the positive control. The resulting tubes were stained with 6 μM Calcein AM (Abcam, ab141420) at 37 °C for 15–30 min and observed under a fluorescence microscope.

### Matrigel plug assay for vascular formation in vivo

Cells (1 × 10^6^ for HUVEC, 3 × 10^6^ for iPSC-ECs) were resuspended in 25 μL basal medium containing 200 ng bFGF and mixed with 100 μL ice-cold Matrigel matrix for subcutaneous injection per mouse. Twelve female nude mice were used in this experiment (3 mice for each group and repeated twice). After 7 days, the plugs were harvested, fixed in 4% formaldehyde in PBS, and embedded in paraffin after dehydration. H&E and anti-hCD31 antibody (Servicebio, GB6017) immunohistochemical (IHC) staining was used to detect formation of vessel-like networks.

### Isolation of PBMCs and NK cells

We obtained blood from HLA-A*11:01 positive healthy donors. PBMCs were isolated from peripheral blood with a density gradient technique (Ficoll, Axis-Shield) and cultured in RPMI 1640 supplemented with 10% human serum (Access Biologicals LLC) and 50 U/mL IL-2. NK cells were enriched from peripheral blood with RosetteSep™ Human NK Cell Enrichment Cocktail (Stemcell, 15,025) and cultured in RPMI 1640 supplemented with 10% human serum.

### CFSE-labeled T cell proliferation assay

iPSC-ECs, used as target cells, were seeded in 48-well plates at 5 × 10^4^ cells per well and treated with 100 ng/mL human IFN-γ (Peprotech, 300-02) for 48 h. On day 0 of co-incubation, PBMCs were labeled with CFSE (Invitrogen, C34554) following the manufacturer’s instructions. Adherent ECs were washed twice with PBS and co-incubated with 2.5 × 10^5^ CFSE-labeled PBMCs in T cell culture media supplemented with 20 U/mL IL-2 and 25 ng/mL IFN-γ. Cells were cultured for 6 days and then stained with anti-huCD3-APC (Biolegend, 317318), anti-huCD4-PE/CY7 (Biolegend, 317414) and anti-huCD8-APC/Cy7 antibodies (Biolegend, 344714) for 20–30 min on ice and analyzed on a FACS CantoII (BD) for CFSE intensity. PBMCs cultured for 6 days without target cells were used as a negative control, while ones treated with 50 ng/mL purified anti-huCD3 antibody (Biolegend, 300402) and 50 ng/mL purified anti-huCD28 antibody (Biolegend, 302902) for 6 days served as a positive control.

### NK cell degranulation assay

5 × 10^4^ iPSC-ECs were seeded in 96-well plates and 24 h later washed once with PBS. 5 × 10^4^ freshly isolated NK cells were added into the wells in NK cell medium supplemented with anti-huCD107a-APC (Biolegend, 328620) and Protein Transport Inhibitor (Monensin) (BD Biosciences, 554724) and spun at 2,000 rpm for 5 min to achieve sufficient effector-target contact. After 20-h co-incubation, cells were stained with anti-huCD56-PE (Biolegend, 318305) for analysis of CD107a cell surface expression. NK cells cultured without target cells were used as negative control, while ones treated with 50 ng/mL PMA (phorbol 12-myristate-13-acetate) (Biogems, 1652981) and 1 μg/mL ionomycin (Biogems, 5608212) were used as positive control.

### NK cell killing assay

4 × 10^4^ iPSC-ECs (target cells) and NK cells (effector cells) at the indicated effector/target ratios were co-incubated in 200 μL NK cell medium in 96-well U bottom for 20 h. One hundred and twenty microliters of supernatant was collected and analyzed by LDH Cytotoxicity Assay Kit (Beyotime, C0017) following the manufacturer’s instructions. NK cell medium was used as background control. iPSC-ECs cultured alone were used as controls for spontaneous LDH release. Lysed iPSC-ECs at endpoint were used as maximal LDH releasing. NK cell cytotoxicity (%) = (NK-iPSC-ECs co-culture LDH activity − spontaneous LDH activity)/(maximum LDH activity − spontaneous LDH activity) × 100%.

### Re-endothelialization of decellularized valves

Decellularized porcine heart valves (DHVs) were prepared according to a previously reported method [28] with a sequential hydrophile and lipophile solubilization of valve cells. The DHVs were sterilized with 0.1% peracetic acid solution for 3 h and then washed with 1 × PBS on a shaker at 37 °C for 2 days, changing the PBS repeatedly to remove peracetic acid residue. On day 3, iPSC-ECs were seeded onto the surface of DHV at a density of 2 × 10^5^ cells/cm^2^ and incubated in a humidified incubator at 37 °C and 5% CO_2_ for 8 days. The seeded valves were fixed in 4% paraformaldehyde, embedded in paraffin after dehydration, and analyzed using H&E and anti-hCD31 IHC staining.

### Flow cytometry analysis

The parent and modified iPSCs as well as differentiated cells were analyzed by flow cytometry (FCM) with labeled antibodies. Cells were dissociated from plates, passed through a 45-mm cell strainer (BD, Cat. No. 352235) and stained in 100 μL of diluted antibodies for 20–30 min on ice. After adding 7-ADD (Biolegend, 420403), samples were analyzed on a FACS CantoII (BD), and the data were processed using FlowJo software.

### Statistics

Statistical analyses were performed with GraphPad Prism8.0. Statistical significance was calculated using two-way ANOVA or one-way ANOVA followed by Tukey’s multiple comparison test, and differences were considered significant at *p* < 0.05.

## Results

### Identification of stem cell quality and HLA-I expression of iPSC lines

As high-quality iPSC lines have higher genomic stability, better homogeneity, a stronger ability of committed differentiation and greater reproducibility in experiments than lower quality ones, we first assessed the quality of several iPSC lines from different origins cultured at the same conditions by analysis of morphology, genomic stability, and pluripotent potency [29] to select a high-quality iPSC line for HLA modifications. We focused on a group of iPSC subclones derived from urine cells [25].

To assess the genomic stability of these iPSCs, iPSC sublines were first analyzed by qRT-PCR to detect the expression level of Sirt1, p53, Tert and CHK1 [30–33], while their pluripotent statement was detected by the expression of TET1, TET3, OCT4-endo, OCT4 and Nanog [34, 35]. Significantly higher expression of these genes in C55 and C56 lines than other lines indicated these two sublines were superior to others (Additional file 1: Fig. S1).

**Fig. 1:**
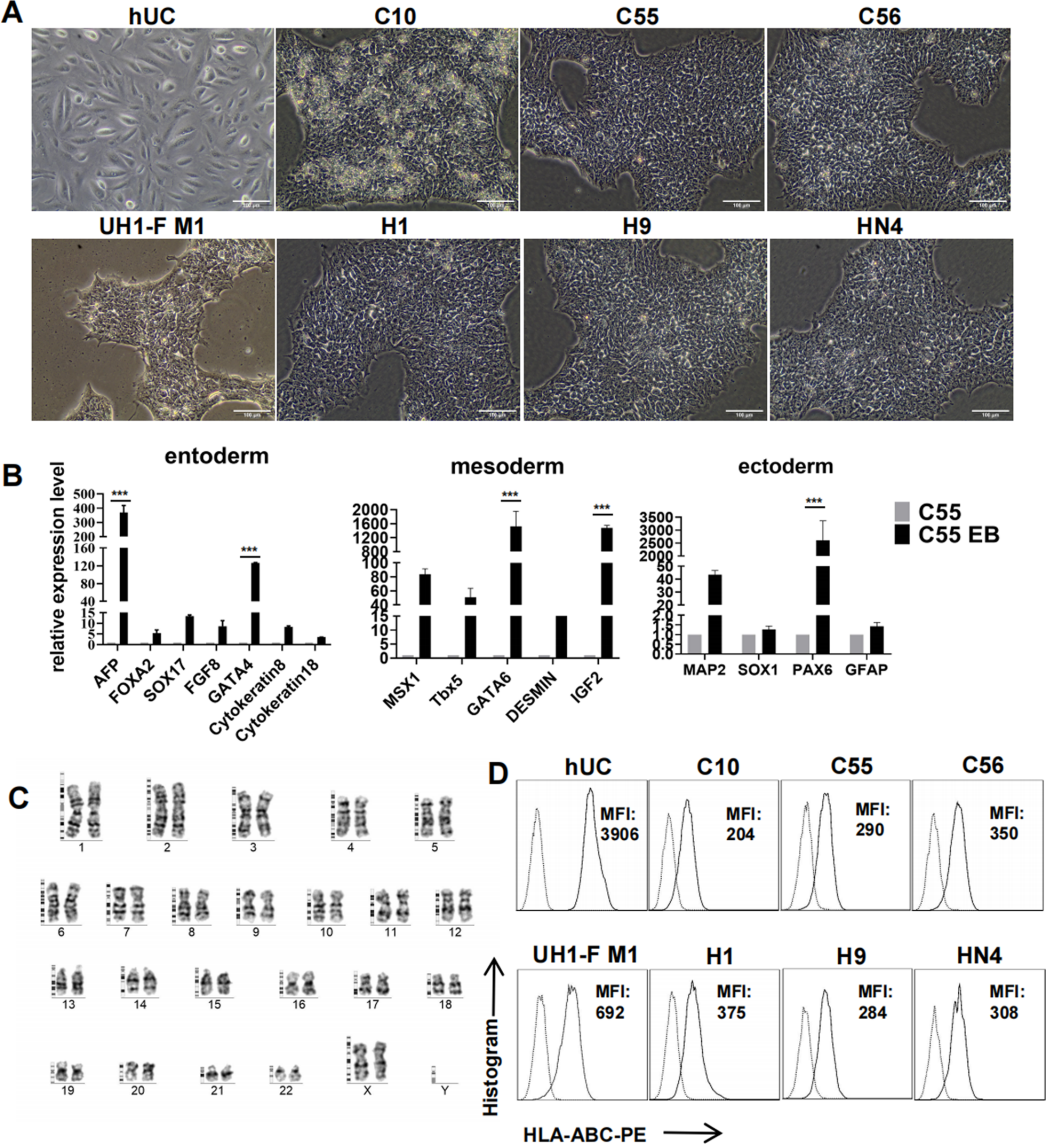
Identification of stem cell quality and HLA-I expression of iPSC lines. **A** Morphology observation of different PSC lines with phase contrast microscope: iPSC lines (C10, C55, C56 and UHI-F MI) and ESC lines (H1, H9 and HN4) were grown in the identical BioCISO medium to compare their undifferentiated morphology, while primary hUCs with epithelioid morphology were used as a differentiated control. Scale bars, 100 µm. **B** Relative expression level of marker genes specific for the three germ layers on the EB differentiation of C55 line by qRT-PCR. The original C55 cells served as undifferentiated control. Relative quantification was normalized to GAPDH (n = 3 replicates). Statistical significance was determined with two-way ANOVA followed by Tukey’s multiple comparison test. Data are means ± SD; ***p < 0.001. **C** G-banding analysis of C55 line showed normal karyotype. **D** FCM analysis of HLA-I expression on the different PSCs. Cells were stained with anti-HLA-ABC-PE (solid line) and isotype control (dotted line) antibodies. MFI of HLA-ABC staining is shown inside the figures

Using ESC lines (H1, H9 and HN4) as undifferentiated reference, we further observed morphology of iPSCs under inverted phase contrast microscope. C55 and C56 sublines were found to maintain a typical undifferentiated cell morphology (tightly packed colonies with defined borders) similar to ESC lines (Fig. 1A). UH1-F M1 and C10 lines showed similar morphological phenotypes but contained a little more detached cells upon the undifferentiated cell layer and very few differentiated fibroblast-like cells (data not shown). The control urinary cells (hUC) showed differentiated epithelioid morphology. These evidences indicate that both of C55 and C56 lines were of higher quality.

C55 line was chosen to examine its pluripotency with in vitro EB formation evaluated via qRT-PCR of marker genes expression of the three germ layers. C55 cells during EB formation upregulated all of these biomarkers in various degrees, which demonstrated that they were able to differentiate into derivatives of all three germ layers (Fig. 1B). In addition, G-banding chromosome analysis of C55 line showed normal karyotype (Fig. 1C). Based on these data, we concluded that C55 line was of pluripotency and normal karyotype.

Prior to the modification of HLA-I gene, we measured HLA-I expression on different PSCs and hUCs via anti-HLA-ABC antibody staining. Mean fluorescence intensity (MFI) of anti-HLA-ABC staining demonstrated that all iPSC/ESC lines expressed weaker HLA-I molecules (MFI from 204 to 692) (Fig. 1D), similar to previous reports [36, 37], and UH1-F M1 cells (MFI = 692) expressed slightly more HLA-I molecules than ESC and urinary cell-derived iPSCs (C55, C56, and C10) (average MFI = 281). The hUC cells expressed the highest level of HLA-I (MFI = 3944) among all examined cell lines.

### Generation of HLA-I gene-modified iPSCs

**Fig. 2:**
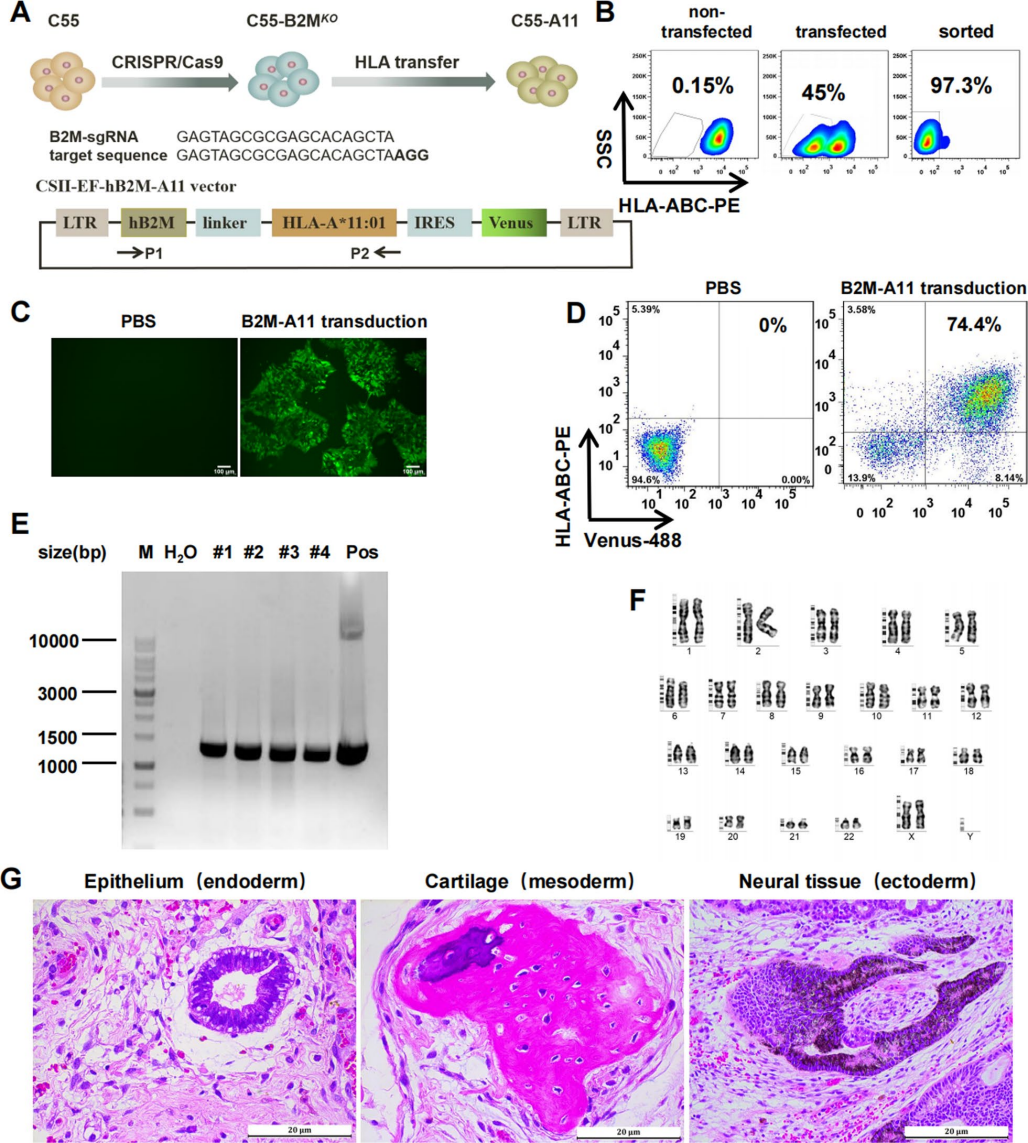
Generation of HLA-exchanged iPSCs. **A** Schematic diagram of the experimental protocol for generation of HLA-exchanged iPSCs via B2M KO and HLA gene transfer. B2M-sgRNA and target sequence are shown in the middle; construction of the CSII-EF-B2M-A11 vector is shown at the bottom. P1 and P2 arrows were primers for identification of HLA gene transfer. **B** B2M targeting of C55 iPSCs by CAS9 gene editing. Non-transfected C55 cells (left panel) were used as HLA-I positive control. The non-transfected (left panel) and transfected (middle panel) cells were stained with anti-HLA-ABC-PE antibody and sorted for HLA-I negative (right panel). **C** HLA gene transfer. Fluorescent images of C55-B2M^*KO*^ cells transducted with CSII-EF-hB2M-A11 vector (right panel) or PBS (left panel). Scale bars, 100 µm. **D** FCM analysis of HLA-I expression. C55-B2M^*KO*^ cells transducted by CSII-EF-hB2M-A11 vector was analyzed by FCM with anti-HLA-ABC-PE staining. The Venus/HLA-I double-positive cells were regarded as the successfully transducted cell population. **E** Identification of HLA gene transfer with specific primers for the CSII-EF-hB2M-A11 vector. The PCR product is approximately 1.3 kb in size. Deionized water was used as the blank control (H_2_O) and the CSII-EF-hB2M-A11 vector was used as a positive control (Pos). **F** Karyotype analysis of the C55-A11#3 line. G-banding of chromosome shows normal karyotype after several rounds of genome manipulations. **G** H&E staining of sections of teratomas generated from C55-A11 in NCG mice. Three typical tissue types from three germ layers are shown. Scale bars, 20 µm

To establish an HLA-exchanging system for iPSCs, we designed first to disrupt HLA-I expression by knocking out *B2M* gene using CRISPR/Cas9 gene editing, and then to express any matched *HLA* allele by lentiviral gene transfer (Fig. 2A).

To knock out *B2M*, the sgRNA was designed to disrupt Exon 1 of *B2M*. The B2M-RNP complex was transfected into iPSCs by Nucleofector electroporation with optimal program for C55 (Additional file 1: Fig. S2). We found that single transfection of B2M-RNP complex resulted in up to 45% of cells losing HLA-I expression (Fig. 2B middle panel). Pure HLA-ABC negative C55 (C55-B2M^*KO*^) were sorted with FACS Aria II sorter (Fig. 2B right panel) for bulk culture and subsequently cloned by LDC. Five out of six clones were HLA-I-negative (Additional file 1: Fig. S3A). Clone #6 (C55-B2M^*KO*^#6) displayed the best growth and was used for subsequent experiments.

To express any matched HLA-I molecules, we designed a B2M-HLA fusion gene to avoid endogenous HLA-I expression. As the HLA-A*11:01 allele is one of the most frequent alleles in southern China [38], we chose it as a model to construct an HLA-I lentiviral expression vector (CSII-EF-hB2M-A11) (Fig. 2A bottom). C55-B2M^*KO*^#6 line was transducted with hB2M-A11 lentiviral supernatant to generate C55-B2M^*KO*^/A11^*Tg*^ (hereafter referred to as C55-A11). The inclusion of the *Venus* gene in the CSII-EF-hB2M-A11 vector allowed us to monitor the transduction under a fluorescence microscope. We found that lentiviral transduction was highly efficient, and that most of the cells showed green fluorescence (Fig. 2C right panel). FCM analysis demonstrated that 74.4% of transduced cells were double positive for Venus and HLA-ABC (Fig. 2D). 4 clones obtained via LDC were analyzed by FCM and PCR technology. All 4 clones were positive for the B2M-A11 fusion gene (Fig. 2E) and express HLA-A11 protein (Additional file 1: Fig. S3B). The clone #3 (C55-A11#3) displaying the best growth was used for subsequent experiments. And karyotype analysis revealed that the C55-A11#3 line had normal chromosome numbers and G-banding (Fig. 2F). Furthermore, we tested cell genetic identity by STR DNA analysis to avoid cell line contamination. Data show that the C55-A11 line was a derivate of the parental C55 line, as they shared the same STR profiling (Additional file 1: Fig. S4).

The pluripotency of the HLA-I-modified C55-A11#3 was tested by an in vivo differentiation assay. H&E staining showed the injected C55-A11#3 cells were able to differentiate into derivatives of all three germ layers; representative tissues such as epithelium, cartilage, and neural tissue are shown in Fig. 2G. These data demonstrated that the HLA-I-modified C55-A11#3 cells maintained pluripotential ability. In conclusion, the karyotypes and pluripotency of the iPSCs were not damaged by the B2M targeting and HLA gene transfer after several rounds of cell and HLA gene manipulations.

### Differentiation of HLA-I-modified iPSCs into endothelial cells

To demonstrate the potential application of HLA-I-modified iPSCs, we attempted to differentiate the C55-A11#3 iPSCs into functional ECs with previous differentiation protocol [27]. The undifferentiated iPSCs were first induced in Priming Medium with BMP4 (an important factor for promotion of mesoderm differentiation) and CHIR99021 (WNT activator/GSK3 inhibitor to enhance expression of mesodermal markers) [39] to commit to mesodermal fate for 3 days, and subsequently replaced with EC Inducing Medium containing hVEGF-A (essential to EC differentiation) and forskolin (a potent cAMP inducer for promoting vascular development) for another 2 days. We first determined the optimal crucial conditions for EC differentiation such as cell plating density and the dose of CHIR99021. 10,000 cells/cm^2^ of plating density and 8 μM CHIR99021 were found to be appropriate for differentiation of C55-A11 (Additional file 1: Fig. S5).

**Fig. 3:**
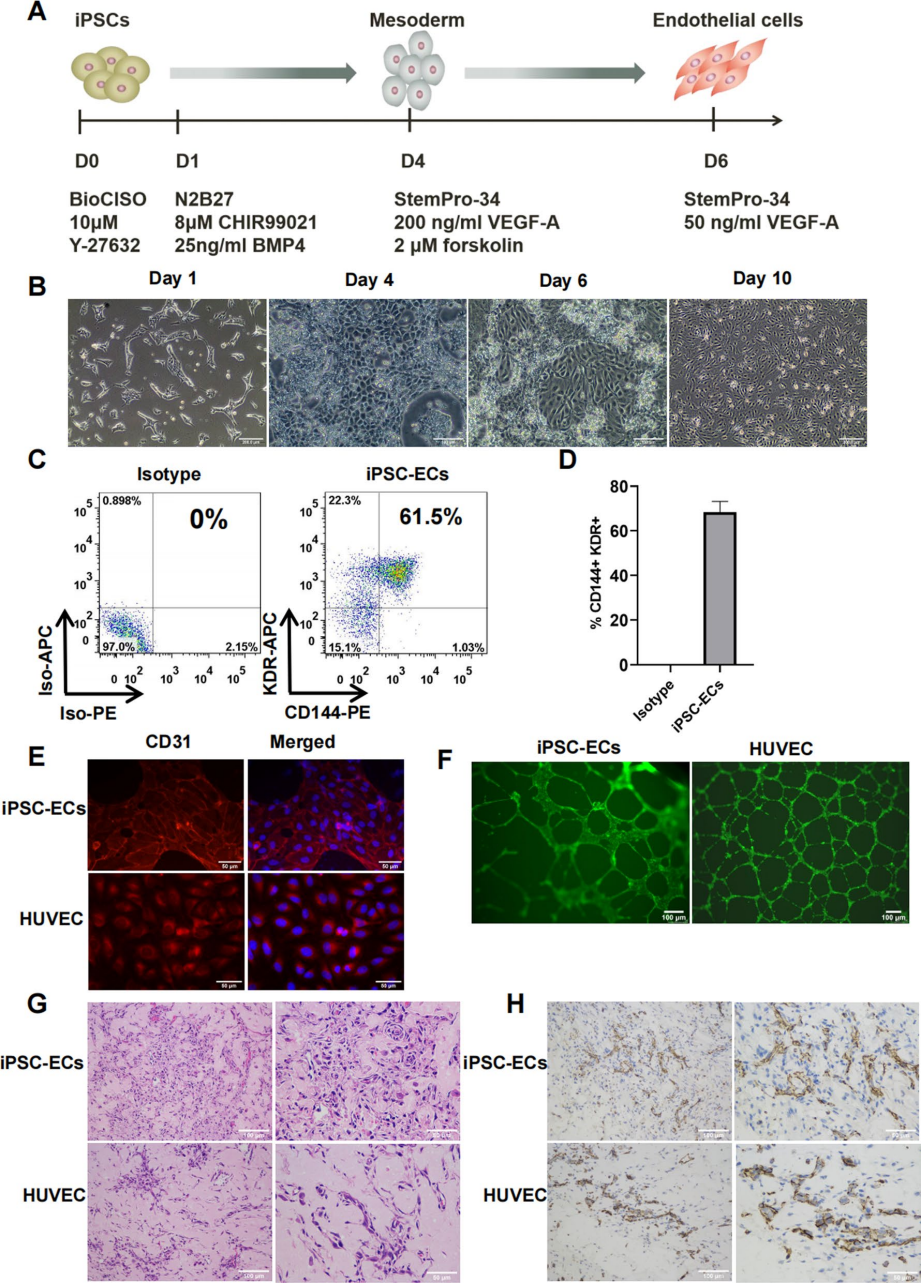
Differentiation of HLA-I-modified iPSCs to endothelial cells. **A** Schematic diagram of the experimental protocol for EC differentiation. **B** The typical phases of EC differentiation shown by phase-contrast imaging. iPSC stage on Day 1, mesoderm stage on Day 4, EC stage on Day 6 and EC expansion stage on Day 10. Scale bars, 200 µm for Day 1 & 10, 100 µm for Day 4 & 6. **C** Representative FCM assay for the KDR and CD144 expression on iPSC-ECs differentiated from C55-A11 iPSCs. Isotype control (left panel) and KDR/CD144 staining (right panel). **D** Histograms displaying the differentiation efficiency of iPSC-ECs from C55-A11. Columns show mean ± SD of 5 independent experiments. ECs were counted by CD144/KDR double positive staining. Isotype staining was used as negative control. **E** Representative immunofluorescent assay for expression of the EC marker CD31 on iPSC-ECs from C55-A11 (upper panel) and positive control HUVECs (lower panel). Left panel: CD31 immunofluorescent staining, right panel: merged imaging of CD31 and DAPI staining. Scale bars, 50 µm. **F** In vitro tube formation assay of iPSC-ECs (left) and HUVECs (right) on Matrigel for 18 h. Images are representative of 5 independent experiments. Scale bars, 100 µm. **G**, **H** In vivo vascular formation in nude mice. Matrigel plugs from C55-A11 iPSC-ECs and HUVEC were stained by H&E dyes (**G**) and anti-CD31 antibody (**H**). Scale bars of G and H, 100 µm (low power) for left panel, 50 µm (high power) for right panel

Using this priming-induction differentiation protocol (Fig. 3A), we observed that C55-A11#3 went through obvious morphological changes during 6-day differentiation (Fig. 3B). On Day 1, cells clustered together after plating in iPSC medium (Fig. 3B, Day 1). From Day 2, cells gradually showed detached morphology and became epithelioid (mesodermal cells) on Day 4 (Fig. 3B, Day 4). On Day 6, cells showed morphological characteristics of ECs, which was a single layer of spindle cells, with obvious nuclei (Fig. 3B, Day 6). After EC differentiation process, the ECs were expanded in StemPro-34 serum-free medium supplemented with 50 ng/mL hVEGF-A for further 4 days, and the cell morphology became more uniform (Fig. 3B, Day 10).

ECs were evaluated by FCM on Day 6 for expression of the endothelial markers KDR (an early EC marker) and CD144 (a late EC marker) [27]. More than 60% (the mean ± SD was 68.38 ± 4.76%) of cells expressed KDR and CD144 markers (Fig. 3C, D). Cells were further analyzed by immunofluorescent assay with a late endothelial marker CD31 [27]. The differentiated and expanded iPSC-ECs were positive for CD31 staining, similar to HUVECs (Fig. 3E).

To analyze the vascular formation of C55-A11 iPSC-ECs, in vitro tube formation assay was first performed to determine the angiogenic potential of iPSC-ECs. Formation of vascular network-like structures of C55-A11 iPSC-ECs and HUVECs was observed within 18 h (Fig. 3F). Network-like structures formed by C55-A11 iPSC-ECs were less than ones from the HUVEC, which might be due to that C55-A11 iPSC-ECs were less mature than the HUVECs. Secondly, to evaluate vascular formation potential in vivo, Matrigel plugs of C55-A11 iPSC-EC or HUVECs were injected into the abdomen side of the nude mice subcutaneously and then were harvested for analysis after 7 days. H&E and anti-huCD31 IHC staining showed vessel-like networks in implants of iPSC-ECs or HUVECs (Fig. 3G, H). These data demonstrated that HLA-I-modified iPSC-ECs had angiogenic potential in vitro and in vivo.

### Reduced T cell responses against allogeneic HLA-I-modified iPSC-ECs in vitro

T cells would be stimulated to proliferate if they contact HLA-I-mismatched allogeneic somatic cells. To investigate whether the single HLA-I matching were able to reduce the immune stimulation to allogeneic T cells, a T cell proliferation assay was conducted to assess the immune compatibility of the HLA-I-modified iPSC-ECs.

**Fig. 4:**
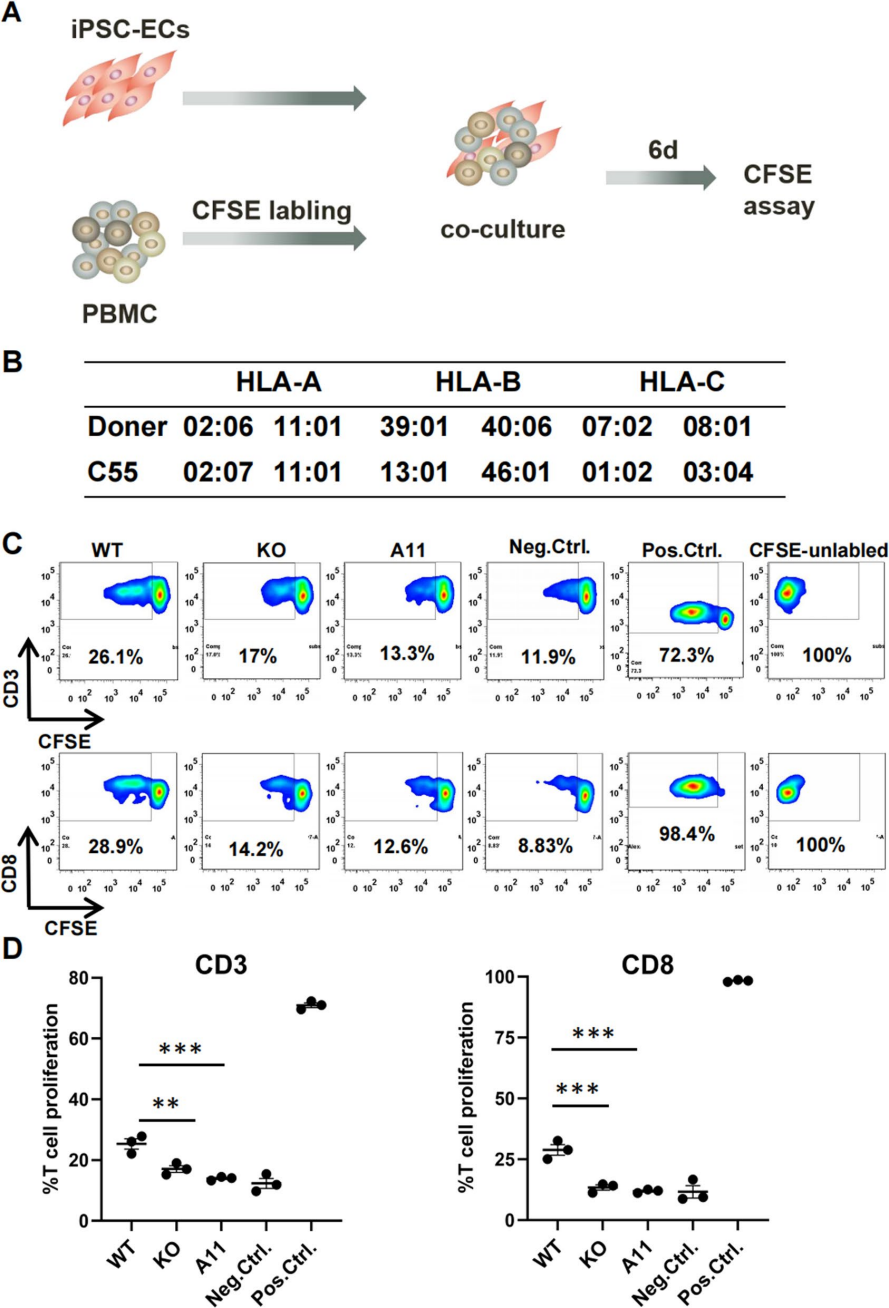
In vitro T cell stimulations by HLA-I-modified iPSC-ECs. **A** Schematic diagram of the protocol for T cell proliferation induced by co-culture of PBMCs with the HLA-I-modified allogeneic iPSC-ECs. **B** HLA genotyping of C55 iPSCs and donor PBMCs. Table shows HLA-I genotyping (A, B and C alleles). **C** T cell proliferation assay with FCM: the percentage of proliferating T cells were plotted by gating of the reduced CFSE fluorescence. CD3+ (upper panel), and CD8+ (lower panel) T cell populations co-cultured with different iPSC-ECs (WT, KO, or A11); the unmodified iPSC-ECs from C55 were called as WT group, B2M KO ones from C55 as KO group and HLA-A11-modified ones from C55-B2M^*KO*^ as A11 group. T cells cultured alone were used as negative controls (Neg.Ctrl.); T cells activated with CD3 and CD28 antibodies served as positive controls (Pos.Ctrl.). **D** Statistical data for T cell proliferation: scatterplots displaying percentage of proliferating T cells in CD3+ (left, *n* = 3 independent experiments) and CD8+ (right, *n* = 3 independent experiments) T cell populations when co-cultured with WT, KO, or A11 iPSC-ECs. Control groups as described in **C**. Statistical significance was determined with paired one-way ANOVA followed by Tukey’s multiple comparison test. Data are means ± SEM; ***p < 0.001, **p < 0.01

Allogeneic PBMCs which have a monoallelic HLA-A*11:01 but mismatched on other alleles (Fig. 4B) were labeled with CFSE and co-cultured with IFN-γ-treated iPSC-ECs derived from the original C55 (WT), C55-B2M^*KO*^ (KO) and C55-A11 (A11) iPSCs at a 5:1 ratio for 6 days. CFSE intensity was measured with FCM in CD3+ T cell and CD8+ T cell subpopulations (Fig. 4A). The gating strategy is shown in Additional file 1: Fig. S6. The percentage of proliferating CD3+ T cells (Fig. 4C, D) in the HLA-I-mismatched WT iPSC-EC group was the highest (25.33 ± 2.92%) among experimental groups; in the KO iPSC-EC group, it reduced to 17.07 ± 1.9%, which was higher than the background level (unstimulated CD3+ cells, 12.36 ± 2.84%). These data demonstrated that only B2M KO still maintained low level of T cell stimulation. When the matched HLA was transferred to C55-A11 iPSC-EC group, it further reduced to 13.97 ± 0.61%, close to the baseline level of unstimulated CD3+ cells. Both of the KO and A11 groups were statistically significant (*p* < 0.01). By analysis of subpopulation, we found that the proliferation inhibition by B2M KO and A11 matching was generally restricted in CD8+ T cells and showed the same trends as CD3+ T cells (Fig. 4C, D). CD4+ T cells had no obvious changes in proliferating cell population among the different groups (data not shown). These results indicated that B2M KO and matched HLA-I gene transfer predominantly inhibited proliferation of the CD8+ T cells rather than the CD4+ T cells. In conclusion, the single HLA-A11-matched iPSC-ECs could significantly prevent CD8+ T cell proliferation induced by HLA-mismatching.

### Reduced NK cell response to HLA-A11-matched ECs

**Fig. 5:**
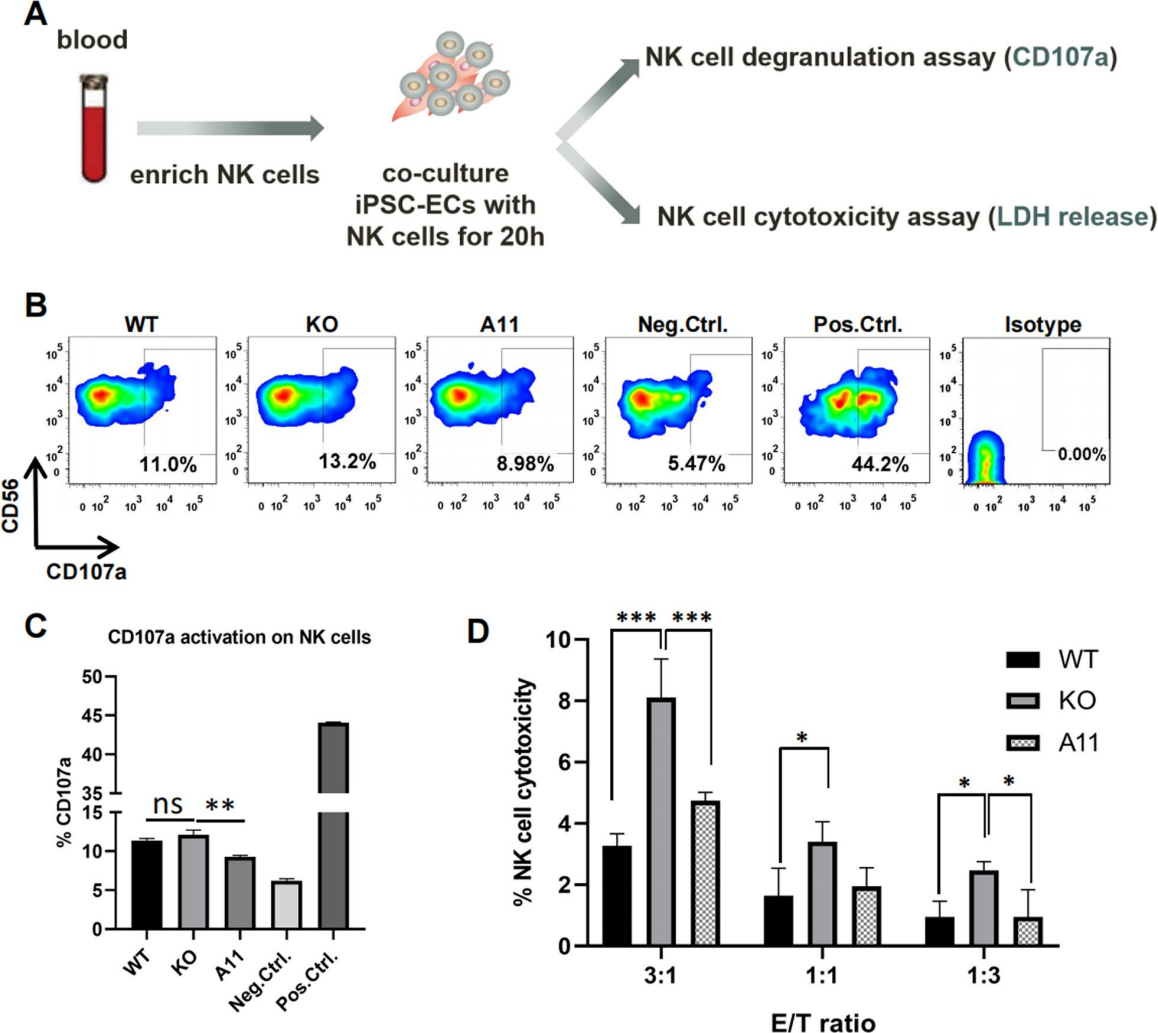
NK cell responses to HLA-I-modified allogeneic iPSC-ECs. **A** Schematic diagram of the experimental protocol for NK cell responses induced by co-culture with the HLA-I-modified allogeneic iPSC-ECs and measured by the degranulation marker CD107a and LDH release. **B** FCM analysis of CD107a on CD56+ NK cells: CD107a+ cells served as a readout for NK cell degranulation against the stimulation of different allogeneic iPSC-ECs. WT, KO and A11 groups described as in Fig. 4C. NK cells cultured alone were used as negative control (Neg.Ctrl); NK cells treated with PMA/ionomycin served as positive control (Pos.Ctrl). **C** Histograms of NK cell degranulation (n = 3 independent experiments). Paired one-way ANOVA followed by Tukey’ s multiple comparison test. Data are mean ± SEM; **P < 0.01. **D** LDH release assay of NK cytotoxicity against WT, KO, and A11 iPSC-ECs: histograms representing the percentage of NK cytotoxicity at the indicated effector/target (E/T) ratios (n = 3 replicates). Unpaired one-way ANOVA followed by Tukey’s multiple comparison test. Data are mean ± SD; *p < 0.05; ***p < 0.001

To analyze whether the single HLA-I matching is sufficient to suppress NK cell activity in co-culture system (Fig. 5A), we first assessed the upregulation of degranulation marker CD107a (LAMP-1) of allogeneic NK cells induced by co-incubation with WT, KO, or A11 iPSC-ECs via FCM analysis of gated CD56+ cells (Additional file 1: Fig. S7). We found that overnight cocultured NK cells reacted slightly higher to B2M^*KO*^ iPSC-ECs (12.1 ± 0.61%) than WT (11.37 ± 0.47%) and significantly higher than A11^*Tg*^ iPSC-ECs (9.28 ± 0.32%) (Fig. 5B, C). Secondly, we further quantify NK cell cytotoxicity to iPSC-ECs by LDH release assay after co-incubation with NK cells. We observed that NK cell cytotoxicity was obviously reduced when NK cells were incubated with A11 iPSC-ECs in different E/T ratios (Fig. 5D). In conclusion, HLA-A11 matching inhibited the NK cell activation induced by B2M KO.

### Re-endothelialization of decellularized valves with HLA-I-modified iPSC-ECs

**Fig. 6:**
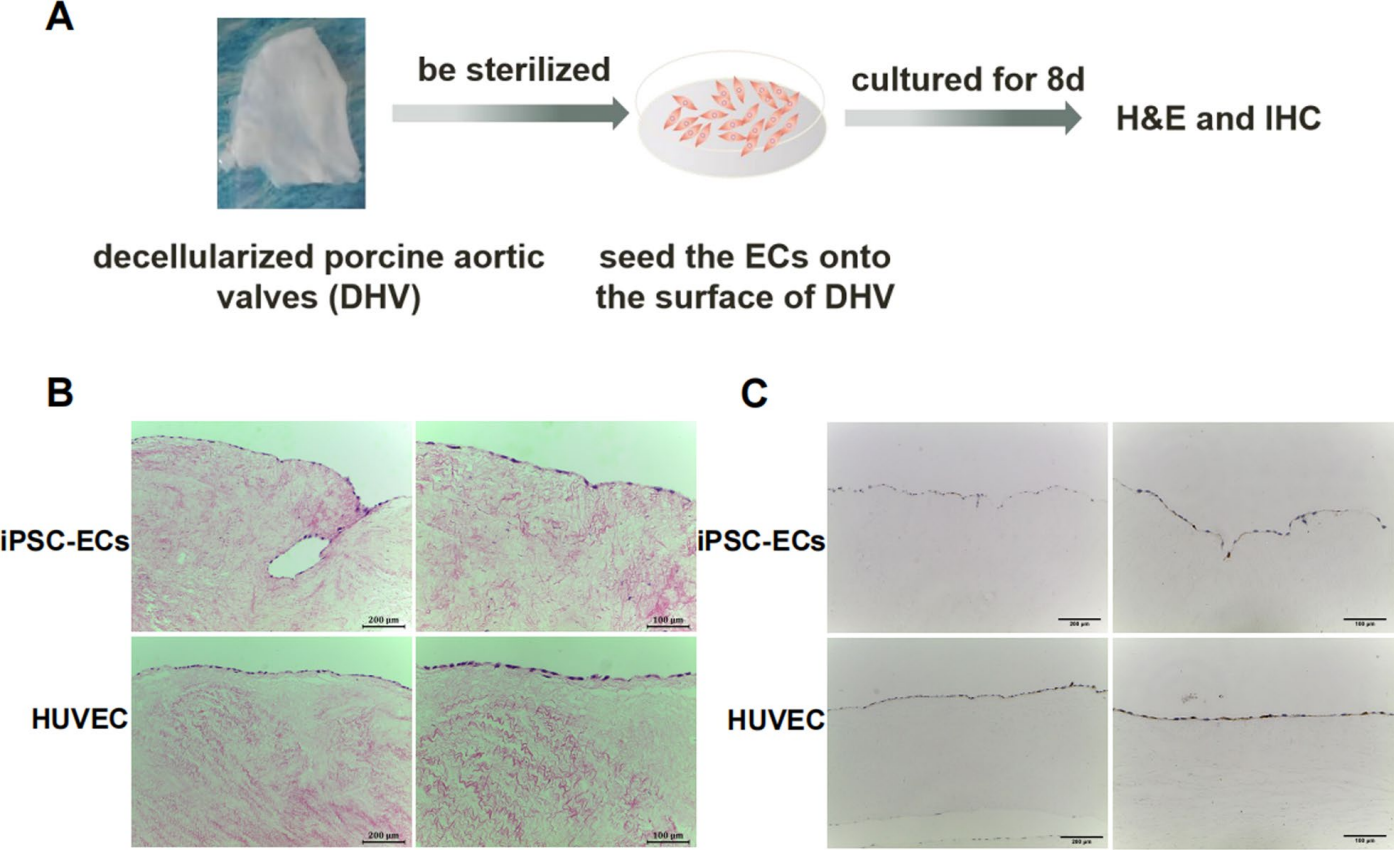
Re-endothelialization of decellularized heart valves with HLA-I-modified iPSC-ECs. **A** Schematic diagram of re-endothelialization of porcine DHV. The DHVs were re-endothelialized by seeding with HLA-I-modified iPSC-ECs from C55-A11 cells or control HUVECs for 8 days and examined with H&E (**B**) and anti-hCD31 IHC (**C**) staining. Scale bars, left panel, 200 µm (low power); right panel, 50 µm (high power)

To explore the possibility of re-endothelialization of biological prosthetic valves with iPSC-ECs, we reconstructed the endothelial layer of the DHV with C55-A11 iPSC-ECs (Fig. 6A). H&E staining showed that the C55-A11 iPSC-ECs formed a thin and single layer of endothelium that completely covered the surface of the valve (Fig. 6B upper panel). Anti-huCD31 IHC staining revealed the cells express the endothelial cell marker CD31 (Fig. 6C upper panel). HUVECs as positive control showed similar staining (Fig. 6B, C lower panels). These experiments demonstrated that HLA-I-modified iPSC-ECs could achieve re-endothelialization of decellularized porcine heart valves.

## Discussion

To solve the problem of NK activation induced by blockage of HLA-I expression via knockout of B2M or HLA-I gene, researchers had generated “universal hypoimmunogenic” iPSCs by gene-editing to knock out allorejection-related genes. Han et al. [40] employed multiplex genome editing to specifically ablate the expression of the highly polymorphic HLA-A/-B/-C via cleavage of HLA-I gene fragments as well as of HLA class II via CIITA KO in PSCs. Subsequently, PD-L1, HLA-G and CD47 were introduced into the safe harbor locus of AAVS1 in these HLA-deficient KO cells to prevent NK-cell-mediated lysis via HLA-G expression and to control macrophage engulfment via overexpressing “don’t-eat me” signal CD47. This approach created an HLA-I/-II double KO hypoimmunogenic iPSCs, however, such all HLA-negative cells are lack of antigen presentation and exist the risk of harboring infected pathogens. To maintain the antigen presentation function of iPSCs, Xu et al. [41] generated single HLA-C-retained iPSCs by disrupting both HLA-A and -B alleles as well as one HLA-C allele. The remaining HLA-C maintains function of antigen presentation and also suppress the NK cell response. However, to establish these “universal hypoimmunogenic” iPSC lines, it needs an amount of complicated manipulation of cell and genome to knockout multiple HLA alleles and exists the risk of off-targets of genome editing.

To overcome disadvantages of previous studies and generate HLA-I-matched iPSCs, we proposed to construct an ‘HLA-exchanging’ technology of allogeneic iPSCs to let HLA-I of iPSCs match to recipient’s alleles for the reduction of the alloresponse. To set up an experimental prototype with one of the commonest HLA alleles HLA-A*11:01 in Chinese population, we established HLA-matching system by knocking out *B2M* and transferring the matched *HLA* genes. *B2M* targeting by single transfection of B2M-RNP resulted in the complete loss of expression of HLA-I in approximately 45% of transfected cells. The lentiviral transduction efficiently transferred the exogenous HLA-A*11:01 gene to 70% iPSCs in one lentiviral infection. HLA-matched iPSCs maintained typical undifferentiated morphology and normal karyotype same to the parent cells during in vitro culture and multiple gene manipulation.

More importantly, the HLA-I-modified iPSCs could be differentiated into derivatives of all three germ layers during in vivo differentiation and committedly into mature ECs in vitro. The angiogenesis assay demonstrated that the iPSC-ECs from HLA-I-modified iPSCs were of angiogenic potential, but not so good as the HUVEC control. Perhaps they were less mature than HUVECs during the short time culture; thus, more efforts are needed to improve the current differentiation method in future. The iPSC-ECs used in our study were not purified, but not observed to form teratoma during experiments. To reduce tumorigenicity from the residual of undifferentiated iPSCs, the iPSC-ECs should be enriched and tested the oncogenicity before clinical applications in following research.

Most importantly, we demonstrated that single HLA-A matching was enough to reduce major allogeneic T cell response and inhibit NK cell activation induced by B2M KO. HLA-I molecules inhibit NK activation via different inhibitory receptors of NK cells, such as HLA-A and HLA-B, respectively, bind to KIR3DL2 and KIR3DL1, HLA-Cs are the ligands of KIR2DL1-3 [42]. Although HLA alleles are highly diverse in human population, the common alleles are quite few. The top 5 of the most common HLA-I alleles (A*02:01, A*01:01, C*07:01, A*03:01 and B*07:02) could cover > 80% of the west population [43], and the top 5 of the most common HLA-I alleles (HLA-A*11:01, HLA-A*24:02, HLA-B*46:01, HLA-C*07:02 and HLA-C*01:02) express in > 90% of the Chinese population [38]. Thus, a small library of iPSC lines modified with single gene from these commonest HLA-I alleles could cover the majority of individuals in world population.

In general, our approach is a direct way to introduce the required HLA alleles into iPSCs without influence on their pluripotency. This method is so simple and effective that it can satisfy the demands of individualized medical services. Tumorigenicity induced by insertional mutagenesis is a concern in our strategy. Previous studies showed that retroviral or lentiviral vector transduction is prone to inducing insertional mutagenesis [44, 45]. In our system, the lentiviral-transducted iPSCs were cloned by limited dilution, and vector insertion into tumor-associated loci should be quite low. Without doubt, our HLA gene matching technology should be improved to transfer the HLA gene into the low-risk original HLA loci in future. Recent studies demonstrated that CRISPR/Cas9 gene editing could efficiently knock in large fragments of genes into selected loci in PSCs and hematopoietic stem cell [46, 47].

Patient HLA-matched iPSCs should have huge potential in individualized regeneration medicine, such as in treating hematopoietic disorders, cardiac valve disease, and retinitis pigmentosa [48]. To demonstrate this possibility, we took ECs as an example to carry out experiments for the improvement of bioprosthetic valves. Bioprosthetic valves lack endothelial cell covering and tend to gradual calcification and thrombosis in vivo compared with mechanical valves [49]. With the priming-induction step-by-step EC differentiation protocol, we efficiently produced iPSC-ECs from ‘HLA-I-matched’ iPSCs and used them to generate re- endothelialization of biological prosthetic valves in short time. The in vivo survival and function of them need further validation in future research.

In the future, both of HLA-I and -II modification according to patients’ HLA genotyping will create individualized “non-immunogenic” allogeneic iPSCs and derivatives for more widespread applications.

## Conclusions

In summary, we successfully created a prototype of HLA-exchanging system for generation of individualized HLA-I-matched allogeneic iPSCs and functional iPSC-ECs with inhibition of alloresponses of T and NK cells. A group of the commonest HLA-I exchanged iPSCs would match more than 95% population. We expect that a small library of ready-to-use HLA-exchanged iPSCs will be widely used for cell therapy in regenerative medicine.

## Supplementary Information

**Additional file 1: Fig. S1.** Heat map of marker gene panels associated with genomic stability for a series of subclones of human iPSC line (LHPb-Yaab) analyzed by q-PCR analysis. Subclones C55 and C56 with a higher level of marker genes are indicated in the red frame. **Fig. S2.** Flow cytometry assay for optimization of Nucleofector program for the C55 cell line. Four different programs (A13, A23, A27, and B16) were tested to transfect C55 cells with the RNP complex of Cas9 protein and B2M sgRNA. The A23 program achieved the best transfection. **Fig. S3.** (A) Flow cytometry assay for six clones of C55-B2M^*ko*^ with anti-huHLA-ABC staining, 5 of them were HLA-I negative. (B) Flow cytometry assay for four clones of C55-A11 with anti-huHLA-ABC staining, all of them were HLA-I positive. **Fig. S4.** STR DNA analysis for C55 and C55-A11, they shared the same STR profiling. **Fig. S5.** Flow cytometry analysis for optimization of CHIR99021 (CHIR)-induced EC differentiation. Five different concentrations (6, 6.5, 7, 7.5, and 8 μM) of CHIR were tested for EC differentiation, which was measured by CD144-PE and KDR-APC staining. **Fig. S6.** Gating strategy of FCM assay used in T cell proliferation. **Fig. S7.** Gating strategy of FCM assay used in NK cell degranulation assay.

## Data Availability

Supporting data are available from the corresponding author upon reasonable request.

## Abbreviations

iPSC: Induced pluripotent stem cell
hUC: Human urine-derived cell
ESC: Embryonic stem cell
EC: Endothelial cells
HUVEC: Human umbilical vein endothelial cell
sgRNA: Single guide RNA
DHV: Decellularized porcine heart valves
