# Correction to: Generation of individualized immunocompatible endothelial cells from HLA-I-matched human pluripotent stem cells

**DOI:** 10.1186/s13287-022-02773-8

**Published:** 2022-02-28

**Authors:** Chanchan Song, Linli Wang, Qingyang Li, Baoyi Liao, Weihua Qiao, Qiang Li, Nianguo Dong, Liangping Li

**Affiliations:** 1 Institute of Clinical Oncology, Research Center of Cancer Diagnosis and Therapy, and Department of Clinical Oncology, The First Affiliated Hospital of Jinan University, Guangzhou, China; 2 Guangzhou Future Homo Sapiens Institute of Biomedicine and Health (GFBH), Guangzhou, China; 3 Guangzhou Regenerative Medicine Research Center, Future Homo Sapiens Institute of Regenerative Medicine Co., Ltd (FHIR), Guangzhou, China; 4 Department of Cardiovascular Surgery, Union Hospital, Tongji Medical College, Huazhong University of Science and Technology, Wuhan, China

## Correction to: Stem Cell Research & Therapy (2022) 13:48 10.1186/s13287-022-02720-7

The original article contained an error in co-author, Linli Wang’s name which has since been corrected.

