# Supplementary material for: Generation of individualized immunocompatible endothelial cells from HLA-I-matched human pluripotent stem cells
Source: Stem Cell Res Ther. 2022 Feb 2;13:48. doi: 10.1186/s13287-022-02720-7 (PMC8812039; doi:10.1186/s13287-022-02720-7)
Supplement: Supplementary file 1 — Additional file 1: Fig. S1. Heat map of marker gene panels associated with genomic stability for a series of subclones of human iPSC line (LHPb-Yaab) analyzed by q-PCR analysis. Subclones C55 and C56 with a higher level of marker genes are indicated in the red frame. Fig. S2. Flow cytometry assay for optimization of Nucleofector program for the C55 cell line. Four different programs (A13, A23, A27, and B16) were tested to transfect C55 cells with the RNP complex of Cas9 protein and B2M sgRNA. The A23 program achieved the best transfection. Fig. S3. (A) Flow cytometry assay for six clones of C55-B2Mko with anti-huHLA-ABC staining, 5 of them were HLA-I negative. (B) Flow cytometry assay for four clones of C55-A11 with anti-huHLA-ABC staining, all of them were HLA-I positive. Fig. S4. STR DNA analysis for C55 and C55-A11, they shared the same STR profiling. Fig. S5. Flow cytometry analysis for optimization of CHIR99021 (CHIR)-induced EC differentiation. Five different concentrations (6, 6.5, 7, 7.5, and 8 μM) of CHIR were tested for EC differentiation, which was measured by CD144-PE and KDR-APC staining. Fig. S6. Gating strategy of FCM assay used in T cell proliferation. Fig. S7. Gating strategy of FCM assay used in NK cell degranulation assay. [file 13287_2022_2720_MOESM1_ESM.docx]

**Supplemental information**

**Fig. S1** Heat map of marker gene panels associated with genomic stability for a series of subclones of human iPSC line (LHPb-Yaab) analyzed by q-PCR analysis. Subclones C55 and C56 with a higher level of marker genes are indicated in the red frame.

**Fig. S2** Flow cytometry assay for optimization of Nucleofector program for the C55 cell line. Four different programs (A13, A23, A27, and B16) were tested to transfect C55 cells with the RNP complex of Cas9 protein and B2M sgRNA. The A23 program achieved the best transfection.

**Fig. S3** (A) Flow cytometry assay for six clones of C55-B2M*^ko^* with anti-huHLA-ABC staining, 5 of them were HLA-I negative. (B) Flow cytometry assay for four clones of C55-A11 with anti-huHLA-ABC staining, all of them were HLA-I positive.

**Fig. S4** STR DNA analysis for C55 and C55-A11, they shared the same STR profiling.

**Fig. S5** Flow cytometry analysis for optimization of CHIR99021 (CHIR)-induced EC differentiation. Five different concentrations (6, 6.5, 7, 7.5, and 8 μM) of CHIR were tested for EC differentiation, which was measured by CD144-PE and KDR-APC staining.

**Fig. S6** Gating strategy of FCM assay used in T cell proliferation.

**Fig. S7** Gating strategy of FCM assay used in NK cell degranulation assay.
